# Validation of Clinical Decision Support Recommendations of a Digital Cognitive Assessment by Expert Cognitive Neurologists

**DOI:** 10.1002/alz.092040

**Published:** 2025-01-09

**Authors:** Ali Jannati, Claudio Toro‐Serey, Joyce Rios Gomes‐Osman, William Isaiah Morrow, Marissa C Ciesla, Russell Banks, David Bates, Sean Tobyne, John Showalter, Alvaro Pascual‐Leone

**Affiliations:** ^1^ Linus Health, Boston, MA USA; ^2^ Harvard Medical School, Boston, MA USA; ^3^ University of Miami Miller School of Medicine, Miami, FL USA; ^4^ Michigan State University, East Landing, MI USA; ^5^ Hebrew SeniorLife, Boston, MA USA

## Abstract

**Background:**

Disease‐modifying treatments for Alzheimer’s disease highlight the need for early detection of cognitive decline. However, most primary care providers do not currently perform routine cognitive testing, in part due to a lack of time and resources to administer and interpret the tests. Brief, self‐scoring, and sensitive digital cognitive assessments, such as the Linus Health Core Cognitive Evaluation (CCE)–which includes the Digital Clock and Recall (DCR™) and the Life and Health Questionnaire (LHQ)–can automatically provide medically‐informed recommendations that can address this need. Here we evaluate the clinical appropriateness of the recommendations generated by this clinical decision support (CDS) tool to guide the diagnosis of cognitive impairment by primary care providers (PCPs).

**Method:**

The CDS tool uses data from the CCE to list potential medical concerns and recommend pathways toward formal diagnosis and/or care. We conducted a retrospective expert‐review study in June 2023 to evaluate the nine CDS pathways for patients aged 55 and above. Experts were five board‐certified cognitive neurologists affiliated with academic institutions. We calculated the median ratings (on a scale of 1 to 9, where 9 is high appropriateness) for the nine CDS pathways across raters. A rating of 7 or above was deemed clinically appropriate.

**Result:**

All 7 pathways related to cognitive impairment received a clinically appropriate rating (median = 7, SD = 0.3, range=7‐8). Pathways below the appropriateness threshold included the one for Green DCR scores (i.e., cognitively unimpaired; median = 6, SD = 0.87) and a preliminary Lecanemab eligibility pathway (median = 5, SD = 1.10).

**Conclusion:**

Pathways and the cognition‐related recommendations generated by the CDS tool of the Linus Health CCE are clinically appropriate, as rated by this initial survey of board‐certified, academic cognitive neurologists. The findings indicate the clinical utility of digital cognitive assessments such as the CCE for guiding the PCPs’ approach to diagnosis and management of patients with cognitive impairment.

